# The impact of generic-only drug benefits on patients' use of inhaled corticosteroids in a Medicare population with asthma

**DOI:** 10.1186/1472-6963-8-151

**Published:** 2008-07-18

**Authors:** Vicki Fung, Ira B Tager, Richard Brand, Joseph P Newhouse, John Hsu

**Affiliations:** 1 Center for Health Policy Studies, Kaiser Permanente Division of Research, Oakland, California, USA; 2 Division of Epidemiology, University of California, Berkeley, USA; 3 Department of Epidemiology and Biostatistics, University of California, San Francisco, USA; 4 Department of Health Care Policy, Harvard Medical School, Boston, Massachusetts, USA; 5 Department of Health Policy and Management, Harvard School of Public Health, Boston, Massachusetts, USA; 6 Kennedy School of Government, Harvard University, Cambridge, Massachusetts, USA

## Abstract

**Background:**

Patients face increasing insurance restrictions on prescription drugs, including generic-only coverage. There are no generic inhaled corticosteroids (ICS), which are a mainstay of asthma therapy, and patients pay the full price for these drugs under generic-only policies. We examined changes in ICS use following the introduction of generic-only coverage in a Medicare Advantage population from 2003–2004.

**Methods:**

Subjects were age 65+, with asthma, prior ICS use, and no chronic obstructive pulmonary disorder (n = 1,802). In 2004, 74.0% switched from having a $30 brand-copayment plan to a generic-only coverage plan (restricted coverage); 26% had $15–25 brand copayments in 2003–2004 (unrestricted coverage). Using linear difference-in-difference models, we examined annual changes in ICS use (measured by days-of-supply dispensed). There was a lower-cost ICS available within the study setting and we also examined changes in drug choice (higher- vs. lower-cost ICS). In multivariable models we adjusted for socio-demographic, clinical, and asthma characteristics.

**Results:**

In 2003 subjects had an average of 188 days of ICS supply. Restricted compared with unrestricted coverage was associated with reductions in ICS use from 2003–2004 (-15.5 days-of-supply, 95% confidence interval (CI): -25.0 to -6.0). Among patients using higher-cost ICS drugs in 2003 (n = 662), more restricted versus unrestricted coverage subjects switched to the lower-cost ICS in 2004 (39.8% vs. 10.3%). Restricted coverage was not associated with decreased ICS use (2003–2004) among patients who switched to the lower-cost ICS (18.7 days-of-supply, CI: -27.5 to 65.0), but was among patients who did not switch (-38.6 days-of-supply, CI: -57.0 to -20.3). In addition, restricted coverage was associated with decreases in ICS use among patients with both higher- and lower-risk asthma (-15.0 days-of-supply, CI: -41.4 to 11.44; and -15.6 days-of-supply, CI: -25.8 to -5.3, respectively).

**Conclusion:**

In this elderly population, patients reduced their already low ICS use in response to losing drug coverage. Switching to the lower-cost ICS mitigated reductions in use among patients who previously used higher-cost drugs. Additional work is needed to assess barriers to switching ICS drugs and the clinical effects of these drug use changes.

## Background

As prescription drug costs continue to rise, public and private payers are increasing insurance restrictions and patient cost-sharing for prescription drugs. Cost-sharing aims to promote cost-effective drug consumption, such as greater use of generic drugs or reduced use of clinically unnecessary drugs, by exposing patients to a greater share of the costs of treatment. For example, in the United States, Medicare Part D drug benefits, which were offered starting in 2006 through a number of private plans, include high levels of cost-sharing. In 2007, about 70% of Part D drug plans included a coverage gap (often referred to as a "doughnut hole") wherein patients lose coverage for all drugs after their total drug spending exceeds $2,400 and until their out-of-pocket costs reach $3,850. About 27% of plans supplement this coverage gap with generic-only coverage, under which patients have coverage for generic drugs, but no coverage for brand-name drugs [1].

Under Medicare Part D cost-sharing arrangements, many beneficiaries may be at risk for losing drug coverage for inhaled corticosteroids (ICS) during the coverage gap, even if they have generic-only coverage supplements, because no generic ICS formulations are available in the U.S. Inhaled corticosteroids are the mainstay of treatment for all forms of asthma, except mild-intermittent [2] and asthma affects approximately seven percent of Americans age 65 years and older [3]. Other medications used similarly to control asthma symptoms, such as bronchodilators and theophyllines, have inferior efficacy and/or safety profiles compared with inhaled corticosteroids [2,4,5]. ICS use is associated with lower rates of emergency department (ED) visits, asthma-related hospitalizations, and mortality [4,6,7].

To gain insight about the impact of brand name drug coverage loss, we examined changes in ICS use among Medicare Advantage (MA) beneficiaries whose plans switched from generic and brand coverage with an annual cap in 2003 to generic-only coverage (i.e., no ICS coverage) in 2004. We compared them with MA enrollees with brand-name coverage in both 2003 and 2004.

## Methods

### Setting

We used automated data from Kaiser Permanente-Northern California (KPNC), an integrated delivery system that provides comprehensive medical care to over three million members, which included about 280,000 MA beneficiaries. Members could join the KPNC MA plan either by purchasing individual insurance or through plans supplemented by their current or former employers. There was only one MA plan (i.e., single cost-sharing structure) for individual subscribers, while employers determined KPNC cost-sharing levels for patients with employer-supplemented plans.

In 2003 individual MA beneficiaries had $10 generic and $30 brand copayments, and a $1,000 benefit cap (meaning they paid full member price for drugs after their total drug expenditures exceeded $1,000 during the year). In 2004, the cap was replaced with generic-only coverage – i.e., patients paid full member price for brand drugs, and $10 copayments for generics throughout the year ("restricted coverage"). By contrast, employer-supplemented MA beneficiaries paid $10 generic and $15–25 brand copayments 2003–2004, with no other limits ("unrestricted coverage"). Patients' coverage type was determined by their employment history and enrollees could not switch between the coverage types (please see Table 1).

**Table 1:** Summary of drug benefits 2003–2004: unrestricted and restricted coverage groups

**Coverage group**	**2003**	**2004**
Unrestricted Coverage	$10 generic copayment	$10 generic copayment
	$15–25 brand copayment	$15–25 brand copayment
Restricted Coverage	$10 generic copayment	$10 generic copayment
	$30 brand copayment	No brand coverage (i.e., full-price for brands)
	$1,000 annual drug benefit cap	No annual drug benefit cap

The full member price includes the drug acquisition and dispensing costs and reflects discounts obtained by the health system; it is generally lower than local retail prices. During the study period, KPNC offered one ICS brand, beclomethasone dipropionate (Qvar), at a significantly lower member price compared with other inhaled corticosteroids. Beclomethasone dipropionate was the most frequently prescribed ICS within the health system, comprising 75% of all ICS prescriptions in our study, and was followed by fluticasone propionate (Flovent), which comprised 23% of ICS prescriptions (i.e., 92% of non-beclomethasone dipropionate prescriptions). The average cost increase due to loss of coverage for a canister of beclomethasone dipropionate was about $15, compared with $65 or $110 for fluticasone propionate 110 mcg or 220 mcg, respectively. The KPNC Institutional Review Board approved the study.

### Study population

All subjects included in this study (N = 1,802) were continuous MA enrollees (January 1, 2003-December 31, 2004), 65+ years old, who met at least one of the following criteria as of January 1, 2003: (1) ≥ 1 inpatient admissions with a primary asthma diagnosis in the previous three years; (2) ≥ 1 ED visits with an asthma diagnosis in the previous two years; or (3) ≥ 2 outpatient visits with an asthma diagnosis in the previous two years. Further, all subjects were dispensed ≥ 30 days-of-supply of an ICS in both 2002 and 2003. We excluded patients with inpatient or outpatient chronic obstructive pulmonary disorder (COPD) diagnoses: ICD-9-CM codes 491 (chronic bronchitis), 492 (emphysema), 493.20 (asthma with COPD), or 496 (COPD) in 2003–2004. We also excluded patients who were dispensed ipratropium bromide (Atrovent), the primary pharmacologic therapy for COPD within KPNC in 2003 or 2004, and patients with Medicaid because they had substantially lower cost-sharing.

### Levels and type of ICS use

We measured ICS use based on days-of-supply dispensed. Days-of-supply were calculated for each prescription by multiplying the number of actuations per canister by the number of canisters dispensed and dividing by the prescribed dose (actuations) and frequency of administration. We carried over remaining supply from month-to-month and calculated patients' ICS supply in each month (standardized to a 30-day month) and year. We considered beclomethasone dipropionate as "lower-cost", and all other ICS brands as "higher-cost". We calculated monthly and annual days-of-supply for lower- and higher-cost ICS drugs separately. We also classified patients as either lower- or higher-cost ICS users in each year by the type with the greater days-of-supply.

### Covariates

Comparisons between the restricted and unrestricted coverage groups were adjusted for a number of potential confounders including age, gender, and race/ethnicity. Race/ethnicity was determined based on routine member surveys and inpatient records (we included an unknown category with 7.5% of subjects). We constructed a neighborhood socioeconomic status (SES) indicator using patients' residential addresses and information from the 2000 US Census. Neighborhoods were considered low SES if ≥ 20 percent of residents had average household incomes below the federal poverty level, or ≥ 25 percent of residents ≥ 25 years old had less than a high school education [8,9]. We adjusted for comorbidity based on the prospective diagnostic cost group (DxCG) score, which is similar to the risk-adjustment method used by the Centers for Medicare and Medicaid Services [10], and included indicators for coronary artery disease, diabetes, heart failure, and hypertension based on membership in the health system's chronic disease registries.

To adjust for asthma severity, we used a number of medication- and diagnosis-related variables in 2002: number of rescue medication canisters dispensed, having a high-dose ICS, any oral steroid dispensed, a high-risk asthma registry flag (see Table 2 footnote for criteria), and any asthma-related ED visits or hospitalizations. We also adjusted for ICS days-of-supply in 2003. We determined the high-dose thresholds based on the Global Initiative for Asthma treatment guidelines [2].

**Table 2:** Study population: patient characteristics

	**Total**	**Unrestricted Coverage**		**Restricted Coverage**	
**Characteristic**		Lower-cost ICS User in 2003	Higher-cost ICS User in 2003	Lower-cost ICS User in 2003	Higher-cost ICS User in 2003
**No. subjects**	1,802	293	175	847	487
Female*	67.7%	63.8%	65.7%	68.8%	68.8%
Age: 65–74	63.5%	60.4%	57.7%	62.6%	69.2%
75–84	31.4%	33.8%	38.9%	31.6%	26.7%
85+	5.1%	5.8%	3.4%	5.8%	4.1%
Race/ethnicity*: White	68.1%	71.0%	70.3%	67.1%	67.4%
Black	4.4%	7.2%	6.3%	2.8%	4.9%
Hispanic	6.9%	3.1%	2.9%	8.5%	8.0%
Asian	10.8%	10.6%	12.6%	10.5%	10.9%
Other	3.2%	3.8%	3.4%	3.1%	3.1%
Unknown	6.5%	4.4%	4.6%	8.0%	5.8%
Neighborhood SES*: Non-low	80.4%	84.0%	83.4%	78.3%	80.9%
Low	17.7%	14.3%	14.9%	19.5%	17.7%
Unknown	1.9%	1.7%	1.7%	2.2%	1.4%
Coronary Artery Disease	10.9%	11.3%	12.6%	10.6%	10.5%
Diabetes	15.5%	14.0%	15.4%	15.9%	15.6%
Heart Failure*	5.7%	8.2%	6.9%	5.4%	4.1%
Hypertension	55.9%	61.8%	55.4%	56.9%	50.7%
Exceeded $1,000 drug cap in 2003	--	--	--	21.3%	44.2%

	**Mean (SD)**	**Mean (SD)**	**Mean (SD)**	**Mean (SD)**	**Mean (SD)**

Comorbidity (DxCG) score*	0.94 (0.57)	1.00 (0.65)	1.02 (0.60)	0.94 (0.57)	0.89 (0.51)

Notes: Lower-cost ICS Users in 2003 defined as patients with greater use of lower-cost than higher-cost ICS drugs in 2003. The 2003 diagnostic cost group (DxCG) score was calculated based on prior year diagnoses and procedures [DxCG Inc., Waltham, MA]. The range for the DxCG score was 0.24 to 5.48, median = 0.79.

* p < .05 for difference between unrestricted and restricted coverage

### Analyses

To adjust for potential confounders, we represented the covariates via a propensity score [11]. For each subject we calculated their probability of having generic-only coverage in 2004 with a logistic model that included all of the covariates described above. Based on these predicted probabilities, we classified patients into propensity score quintiles [12]. We confirmed that covariate distributions were comparable across coverage groups within each quintile and found no statistically significant differences using Pearson chi-square tests for categorical variables and t-tests for continuous variables. As a sensitivity test, we compared propensity score with traditional covariate adjustment; point estimates and standard errors were similar across the methods.

To examine *monthly *ICS use we used a linear model and estimated coefficients using a generalized estimating equation (GEE) approach. The models included indicators for propensity score quintile (2–5), month (2–24), restricted coverage, and interactions between restricted coverage and month. The coefficients of the interactions test the hypothesis that coverage type was associated with different patterns of ICS use over months.

To examine changes in *annual *ICS use associated with restricted coverage we calculated the change in days-of-supply (2004 minus 2003) for each subject and used this difference as the outcome in linear models, adjusted for propensity score quintile. We examined annual changes among all subjects, among users of higher- and lower-cost ICSs in 2003, separately, and by type of ICS use in 2004, separately. We also examined changes in ICS use among patients identified as having high-risk asthma in the health system's disease registry, and those with lower-risk asthma (i.e., no high-risk flag).

Lastly, we examined the patient characteristics associated with having no ICS use in 2004 using multivariable logistic regression, and included the covariates described above.

## Results

### Patient characteristics

Table 2 displays the characteristics of the 1,802 study subjects: 1,334 subjects (74%) had restricted, and 468 (26.0%) had unrestricted coverage. As shown in Table 3, higher-cost ICS users in 2003 were more likely to have high-risk asthma, prior oral steroid use, and a high ICS dose level, compared with lower-cost ICS users. In 2003, the average ICS days-of-supply dispensed was 187.5 (supply for 51.4% of days). In 2004, use decreased for all groups; overall, patients had an average of 161.3 days-of-supply (supply for 44.2% of days).

**Table 3:** Study population: asthma characteristics

		**Unrestricted Coverage**		**Restricted Coverage**	
Characteristic	Total	Lower-cost ICS User in 2003	Higher-cost ICS User in 2003	Lower-cost ICS User in 2003	Higher-cost ICS User in 2003
**No. subjects**	1,802	293	175	847	487
High-risk asthma flag in 2002*	12.1%	10.9%	17.1%	9.0%	16.4%
ED visit or hospitalization for asthma in 2002	6.7%	9.2%	5.1%	5.3%	8.2%
Oral steroid Rx dispensed in 2002	31.4%	27.7%	32.6%	27.5%	39.8%
Low ICS dose level in 2002	31.4%	55.3%	10.9%	53.5%	14.0%
Medium ICS dose level in 2002	36.2%	36.5%	37.1%	36.6%	35.1%
High ICS dose level in 2002	24.8%	8.2%	52.0%	9.9%	50.9%

	Mean (SD)	Mean (SD)	Mean (SD)	Mean (SD)	Mean (SD)

No. of rescue medication canisters dispensed in 2002	3.73 (4.79)	3.57 (4.80)	4.06 (5.10)	3.44 (4.12)	4.18 (5.64)
ICS Days-of-supply in 2003**	187.5 (103.3)	200.0 (107.2)	204.4 (104.3)	187.8 (102.7)	173.4 (99.9)
ICS Days-of-supply in 2004**	161.3 (120.3)	184.7 (124.7)	184.4 (119.0)	162.0 (120.9)	137.5 (112.8)

Notes: Lower-cost ICS Users in 2003 defined as patients with greater use of lower-cost than higher-cost ICS drugs in 2003. ICS dose level categorized based on the first prescription in 2002 and the Global Initiative for Asthma treatment guidelines.

* The registry classified patients as high-risk if they met any of the following criteria: (1) ≥ 1 inpatient admissions with a primary asthma diagnosis in the previous 12 months, (2) ≥ 1 inpatient admissions with a secondary asthma diagnosis in the previous 12 months, accompanied by a primary asthma-related diagnosis, (3) ≥ 1 ED visits with an asthma diagnosis in the previous six months, (4) ≥ 5 prescriptions for asthma medication in the previous six months with ≥ 2 oral steroid prescriptions in the previous 12 months, or (5) ≥ 12 canisters of inhaled beta-agonists in the previous 12 months.

** p < .05 for difference between unrestricted and restricted coverage groups

### Monthly ICS use

**Figure 1:**
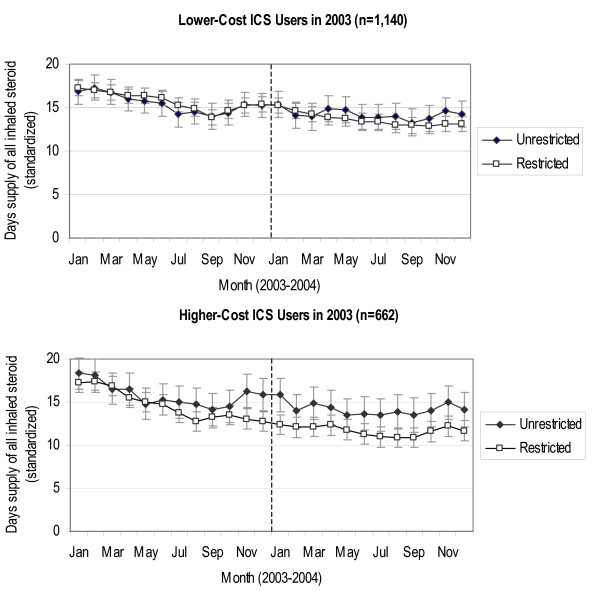
**Adjusted mean monthly ICS days-of-supply (2003–2004) for lower-cost and higher-cost ICS users in 2003 by coverage group**. Note: The graph presents the adjusted mean monthly days-of-supply (standardized to a 30-day month) of all inhaled corticosteroids for the Unrestricted and Restricted Coverage groups. Bars represent 95% confidence intervals.

Figure 1 presents monthly ICS use separately for lower- and higher-cost ICS users with restricted and unrestricted coverage. Among lower-cost ICS users in 2003, there was little difference in ICS use between the coverage groups throughout the study period. Among higher-cost ICS users in 2003, restricted coverage patients decreased their ICS use relative to unrestricted coverage patients toward the end of 2003, as an increasing number of restricted coverage subjects exceeded the $1,000 benefit cap (Table 2), and these differences remained in 2004 after the loss of brand-name coverage.

### Changes in ICS type

**Figure 2:**
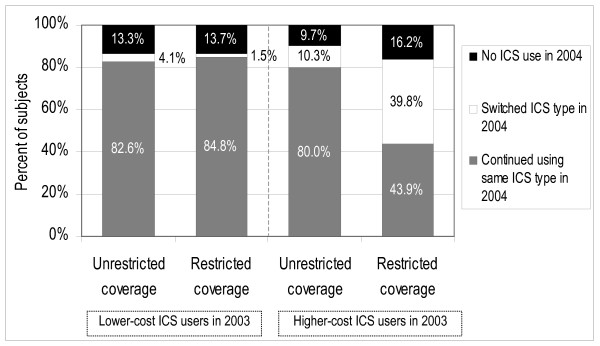
**Type of ICS use in 2004 by coverage group and type of ICS use in 2003**. Note: Lower-cost ICS Users in 2003 and 2004 were defined as patients with greater use of lower-cost than higher-cost ICS drugs in each year, and vice-versa. Patients were defined as having No ICS Use in 2004 if their total annual ICS days-of-supply was zero.

Figure 2 displays the distribution of the type of ICS use in 2004 (lower-cost versus higher-cost), by patients' type of ICS use in 2003 and coverage group. Overall, 13.9% of subjects had no ICS use in 2004. Among higher-cost ICS users in 2003, 16.2% of restricted and 9.7% of unrestricted coverage patients had no ICS use in 2004 (p = 0.036). In addition, 39.8% of restricted versus 10.3% of unrestricted coverage patients switched to the lower-cost ICS in 2004 (p < .001). Among lower-cost ICS users in 2003, levels of non-use of ICS drugs in 2004 were similar for unrestricted and restricted coverage patients; switching to higher-cost ICS drugs was uncommon in both groups, but higher in the unrestricted coverage group (4.1% versus 1.5%, p = 0.010).

### Annual changes in ICS use

Table 4 presents the associations between coverage type and changes in ICS use from 2003 to 2004, adjusted for covariables. Overall, restricted coverage patients reduced their ICS use by an average of 15.5 days-of-supply (95% confidence interval (CI): -25.0 to -6.0) more than unrestricted coverage patients. Among lower-cost ICS users in 2003, restricted coverage was associated with a reduction of 12.8 days-of-supply (CI: -24.3 to -1.3); among higher-cost ICS users in 2003, restricted coverage was associated with a reduction of 21.8 days-of-supply (CI: -38.5 to -5.1).

**Table 4:** Adjusted change in annual ICS days-of-supply for lower and higher-cost ICS users in 2003: restricted vs. unrestricted coverage

	**Change in ICS Days-of-Supply from 2003 to 2004**		**Change in ICS Days-of-Supply from 2003 to 2004: Restricted vs. Unrestricted Coverage (Difference-in-difference)**		
	Restricted Coverage	Unrestricted Coverage	Restricted vs. Unrestricted	(95% CI)	p-value
**All Subjects (n = 1,802)**	-30.27	-14.81	-15.47	(-24.98, -5.95)	0.001
**Lower-cost ICS Users in 2003 (n = 1,140)**	-26.38	-13.55	-12.84	(-24.34, -1.33)	0.029
Continued using lower-cost ICS in 2004 (n = 960)	-13.08	0.63	-13.71	(-25.52, -1.90)	0.023
Switched to higher-cost ICS in 2004 (n = 25)	-54.24	29.51	-83.75	(-171.58, 4.07)	0.060
**Higher-cost ICS Users in 2003 (n = 662)**	-37.40	-15.60	-21.80	(-38.46, -5.13)	0.010
Continued using higher-cost ICS in 2004 (n = 354)	-39.81	-1.19	-38.62	(-56.96, -20.28)	< .001
Switched to lower-cost ICS in 2004 (n = 212)	0.71	-18.04	18.75	(-27.46, 64.96)	0.425

Notes: Each row represents a separate OLS regression model. Among lower-cost ICS users in 2003, 155 subjects had no ICS drug use in 2004, among higher-cost ICS users in 2003, 96 subjects had no ICS drug use in 2004. We calculated mean changes in ICS days-of-supply for each coverage group by using the *lincom *command in Stata 8.2 and the mean levels of the covariables.

For higher-cost ICS users in 2003, the effect of restricted coverage on ICS use differed by the type of medication used in 2004. Restricted coverage was not associated with decreased ICS use (2003–2004) among patients who switched to the lower-cost ICS in 2004 (18.7 days-of-supply, CI: -27.5 to 65.0), but was among patients who did not switch (-38.6 days-of-supply, CI: -57.0 to -20.3).

We also examined changes in ICS use associated with restricted coverage, stratified by asthma disease severity, as defined by having a high-risk asthma flag in the disease registry (Table 5). Table 5 also presents levels of ICS use in each group in 2003. As expected, baseline ICS use was greater among higher-risk asthma patients as compared with lower-risk within each of the coverage groups; however, differences by severity level were relatively small among patients with coverage restrictions as compared with those without restrictions in either year (difference of 11.6 vs. 29.9 days-of-supply). In contrast, restricted coverage was associated with similar reductions in ICS use in the higher-risk (-15.0 days-of-supply, CI: -41.4 to 11.4) and lower-risk (-15.6 days-of-supply, CI: -25.8 to -5.3) asthma groups.

**Table 5:** Adjusted change in annual ICS days-of-supply for lower and higher-risk asthma patients: restricted vs. unrestricted coverage

	**Baseline ICS Days-of-Supply (2003)**		**Change in ICS Days-of-Supply from 2003 to 2004**		**Change in ICS Days-of-Supply from 2003 to 2004: Restricted vs. Unrestricted Coverage (Difference-in-difference)**		
	Restricted Coverage	Unrestricted Coverage	Restricted Coverage	Unrestricted Coverage	Restricted vs. Unrestricted	(95% CI)	p-value
Lower-risk asthma patients (n = 1,584)	181.20	197.70	-30.46	-14.90	-15.56	(-25.77, -5.34)	0.003
Higher-risk asthma patients (n = 218)	192.79	227.58	-28.97	-14.00	-14.97	(-41.38, 11.44)	0.265

Notes: Each row represents a separate OLS regression model. We classified those with a high-risk asthma flag as higher-risk and those with no high-risk flag as lower-risk asthma patients (please see Table 3). We calculated mean changes in ICS days-of-supply for each coverage group by using the *lincom *command in Stata 8.2 and the mean levels of the covariables. The baseline ICS days-of-supply presents the unadjusted mean days-of-supply in each group in 2003.

### Characteristics associated with no ICS use in 2004

In separate analyses (more details available upon request), among all subjects and among higher-cost ICS users in 2003, restricted coverage was not significantly associated with having no ICS use in 2004 (OR = 1.18, CI: 0.83–1.67; and OR = 1.65, CI: 0.89–3.04, respectively). Among all subjects, having a high-dose ICS in 2002 and greater ICS use (days-of-supply) in 2003 were associated with lower odds of no ICS use in 2004 (OR = 0.65, CI: 0.46–0.92; and OR = 0.89, CI: 0.87–0.91, respectively).

## Discussion

Inhaled corticosteroids are a critical component of therapy for persistent asthma. In this population of Medicare beneficiaries with asthma, levels of ICS use were low (as shown in Table 3): on average, patients had drug supply for only about half of the year in 2003. Moreover, loss of coverage for ICS drugs in 2004 was associated with reductions in ICS use.

Restricted drug coverage was associated with a modest reduction in ICS use even among patients who faced smaller increases in out-of-pocket costs because they were using the lower-cost ICS in 2003 before the loss of brand-name coverage. Changes in ICS use from 2003 to 2004 were more pronounced among the one-third of patients who were prescribed higher-cost inhaled corticosteroids in 2003. As expected, a number of these patients with restricted coverage switched to the lower-cost drug in 2004 (about 40 percent), and switching appeared to mitigate reductions in 2004 ICS use due to loss of drug coverage. However, many patients did not switch to the lower-cost ICS and those with restricted coverage reduced their ICS use by over one month of supply compared with patients with unrestricted coverage.

Patients who were taking higher-cost ICS drugs at baseline exhibited greater asthma severity compared with lower-cost ICS users, as indicated by several medication and diagnoses measures shown in Table 3. In addition, in analyses stratified by asthma severity level (Table 5), loss of brand-name drug coverage was associated with reductions in ICS use, even among those with a strong clinical indication for the drug therapy (i.e., higher-risk asthma patients). Decreases in ICS use in the high-risk asthma group may reflect greater increases in drug costs associated with the loss of coverage, due to higher average daily doses. Alternatively, the high-risk asthma flag indicates a prior history of frequent or serious exacerbations, which may be associated with worse self-management of asthma symptoms. These findings raise important questions about potential adverse clinical consequences of reduced ICS use due to restrictive drug coverage policies, particularly for patients with greater clinical need for ICS therapy. Studies in non-elderly asthma populations have found that lower levels of ICS use are associated with greater hospitalizations, emergency department visits, and oral steroid use [6,13,14]. In addition, evidence suggests that increases in drug costs associated with ICS use are off-set by decreases in other direct medical costs [15-19]. Research is needed to assess clinical outcomes associated with these drug coverage policies, as well as to determine the impact on total medical costs.

Encouraging greater substitution of higher-cost with lower-cost ICS drugs could mitigate potential adverse effects of restricted drug coverage policies. In this setting, switching to the lower-cost ICS would not be likely to result in adverse clinical effects as head-to-head trials have not produced definitive conclusions on the comparative efficacy of different inhaled corticosteroids [5]. There is some evidence that suggests that potency, or the dose necessary to achieve a specific effect, may differ across different ICS types. Notably, fluticasone propionate (the primary higher-cost ICS in this study) appears to achieve similar improvements in lung function at half the dose of beclomethasone dipropionate (the lower-cost ICS) [20,21]. Thus, the higher-cost ICS drugs may offer simpler daily dosing regimens, such as requiring fewer administrations per day, compared with the lower-cost ICS, which might encourage better regimen adherence especially for patients prescribed a high ICS dose (i.e., patients with a more frequent dosing schedule) [22]. More work is needed to explore whether patients were aware of the availability of a lower-cost ICS and to examine other potential barriers to switching.

Poor adherence to ICS therapy has been demonstrated in other studies [4,23-28]. Two Canadian studies found that increases in drug cost-sharing were associated with decreases in ICS use in elderly patients and children [29,30]. To our knowledge, no studies have examined the impact of drug coverage restrictions on ICS use in Medicare populations. Our findings suggest that patients who lose coverage for their inhaled corticosteroids may reduce their levels ICS use. Of importance, this health system was able to provide a lower price on beclomethasone dipropionate, due to negotiated discounts, than is likely to be available in other settings. Therefore, our findings of decreases in ICS use due to generic-only coverage in this setting raise important questions about the potential for more pronounced effects in other settings where a lower-cost ICS is not available. This highlights the importance of providing patients with a low-cost ICS option during uncovered periods, such as Part D coverage gaps, to encourage continued adherence to ICS therapy.

Because ours was a non-randomized study it is possible that differential changes in ICS use between the restricted and unrestricted groups are due to unmeasured differences. We were able to adjust for a large a number of socio-demographic and clinical characteristics, including a number of asthma severity measures. Identifying patients with persistent asthma based on administrative data is challenging; we examined other cohort definitions of varying restrictiveness (e.g., including patients with a secondary inpatient diagnosis of asthma) and found similar results across these definitions. ICS use was measured using pharmacy data. These data do not capture whether patients actually took dispensed medications or took them correctly; we also did not assess the clinical effects of changes in ICS use. The pharmacy data do not include drugs dispensed outside of KPNC; however, patients had a financial incentive to purchase their medications within KPNC because of favorable in-system prices throughout the study period. As a result, in telephone interviews, members report rarely going outside of the system to obtain medications or other services [31]. Lastly, this integrated delivery system may have an enhanced ability to monitor care and control drug costs, which may minimize potential harms due to increases in cost-sharing.

## Conclusion

This study highlights the importance of offering a low-cost option within the ICS drug class, particularly during uncovered periods, such as during the Medicare Part D coverage gap. Insurers may also consider maintaining coverage for ICS drugs during otherwise uncovered periods for patients with a clinical need for these drugs. Additional work is needed to understand patient responses to drug cost-sharing, especially for patients who did not switch to the lower-cost option yet reduced ICS use, as well as the clinical effects of restricted drug coverage policies.

## Abbreviations

CI: confidence interval; COPD: chronic obstructive pulmonary disorder; ED: emergency department; ICS: inhaled corticosteroids; KPNC: Kaiser Permanente-Northern California; MA: Medicare Advantage; OR: odds ratio.

## Competing interests

The authors declare that they have no competing interests.

## Authors' contributions

VF made substantial contributions to the study's conception and design, acquisition of data, analysis and interpretation of data, drafted the manuscript, and revised the manuscript critically for important intellectual content. IBT made substantial contributions to the study's design, analysis and interpretation of the data, and revised the manuscript critically for important intellectual content. RB made substantial contributions to the study's design, analysis and interpretation of the data, and revised the manuscript critically for important intellectual content. JPN made substantial contributions to the study's design, analysis and interpretation of the data, and revised the manuscript critically for important intellectual content. JH made substantial contributions to the study's conception and design, acquisition of data, analysis and interpretation of the data, and revised the manuscript critically for important intellectual content. All authors read and approved the final manuscript.

## Pre-publication history

The pre-publication history for this paper can be accessed here:


